# P-709. Incidence of Hospitalizations for Laboratory-Confirmed RSV, COVID-19, and Influenza among Children under-18 years — Respiratory Virus Hospitalization Surveillance Network (RESP-NET), United States, 2022–2024

**DOI:** 10.1093/ofid/ofaf695.921

**Published:** 2026-01-11

**Authors:** Mila Shakya, Ian D Plumb, Alissa O’Halloran, Dawud Ujamaa, Pam Daily Kirley, Isaac Armistead, Kimberly Yousey-Hindes, Kyle P P Openo, Sarah E Rojewski, Erica Mumm, Caroline McCahon, Grant Barney, Erin Licherdell, Melissa Sutton, Holly B Staten, Monica E P Patton, Catherine Bozio, Fiona P Havers

**Affiliations:** Centers for Disease Control and Prevention, Atlanta, Georgia; Division of Foodborne, Waterborne, and Environmental Diseases, Centers for Disease Control and Prevention, Atlanta, GA, Atlanta, Georgia; CDC, Atlanta, GA; Centers for Disease Control and Prevention, Atlanta, Georgia; California Emerging Infections Program, Oakland, California; Colorado Department of Public Health and Environment, Denver, Colorado; Yale School of Public Health, New Haven, Connecticut; Emory University School of Medicine, Atlanta, Georgia; Michigan Department of Health and Human Services, Oak Park, Michigan; Minnesota Department of Health, Saint Paul, Minnesota; New Mexico Department of Health, Sante Fe, NewMexico; New York State Department of Health, Albany, New York; University of Rochester Medical Center, Rochester, New York; Public Health Division, Oregon Health Authority, Portland, Oregon; Salt Lake County Health Department, Salt Lake City, Utah; Centers for Disease Control and Prevention, Atlanta, Georgia; CDC, Atlanta, GA; Centers for Disease Control and Prevention, Atlanta, Georgia

## Abstract

**Background:**

Respiratory syncytial virus (RSV), SARS-CoV-2, and influenza virus infections are leading causes of severe illness in children. We compared population-based pediatric hospitalization rates for illness associated with these viruses using the Respiratory Virus Hospitalization Surveillance Network (RESP-NET).

**Figure:**
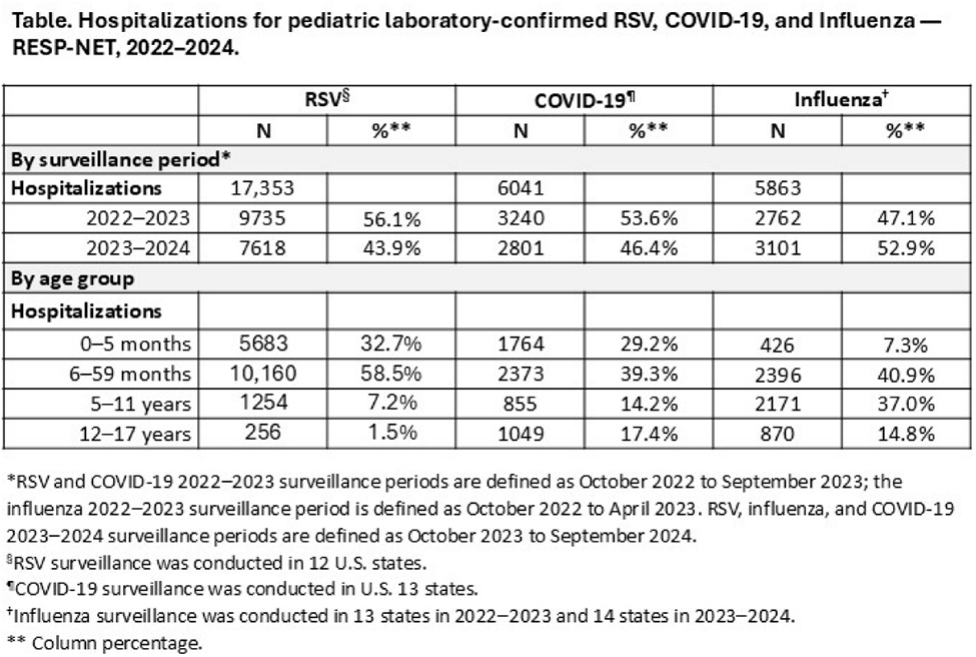

**Figure:**
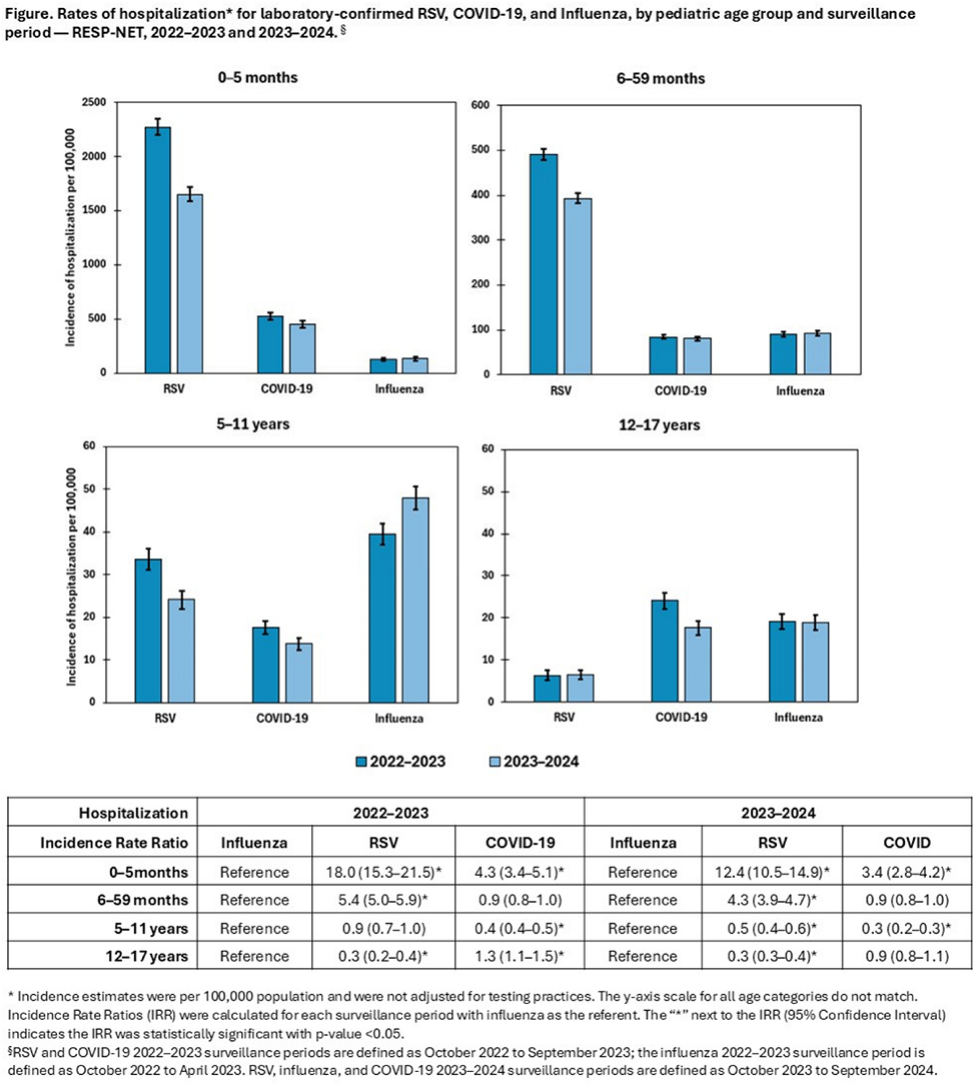

**Methods:**

Children < 18 years hospitalized with RSV, COVID-19, and influenza during October 2022–September 2024 were identified in RESP-NET, which includes patients with positive screening or clinician-directed laboratory test for each virus ≤14 days prior to or during hospitalization at >300 U.S. hospitals. We calculated hospitalization rates by age group and surveillance period, and incidence rate ratios.

**Results:**

During 2022–2024, 15,843/17,353 (91%) hospitalizations with RSV, 4137/6041 (68%) with COVID-19, and 2822/5863 (48%) with influenza occurred in < 5 year-olds (Table); this age group had the highest hospitalization rates (Figure). Among 0–5 month-olds, RSV hospitalization rates during 2022–2023 and 2023–2024 were 18.0 (15.3–21.5) and 12.4 (10.5–14.9) times higher than respective influenza rates; COVID-19 rates were 4.3 (3.4–5.1) and 3.4 (2.8–4.2) times higher. Among 6–59 month-olds, RSV hospitalization rates were 5.4 (5.0–5.9) and 4.3 (3.9–4.7) times higher; COVID-19 rates did not differ statistically from influenza rates. Among 5–11 year-olds, RSV hospitalization rates were 0.5 (0.4–0.6) times influenza rates in 2023–2024, but not statistically different in 2022–2023; COVID-19 hospitalization rates were 0.4 (0.4–0.5) and 0.3 (0.2–0.3) times influenza rates in 2022–2023 and 2023–2024 respectively. Among 12–17 year-olds, RSV hospitalization rates were 0.3 (0.2–0.4) and 0.3 (0.3–0.4) times influenza rates during 2022-2023 and 2022–2024; COVID-19 rates were 1.3 (1.1–1.5) times influenza rates in 2022-2023 but not statistically different in 2022–2024.

**Conclusion:**

RSV accounted for the highest hospitalization rates among infants and young children. Among children 5–17 years, hospitalization rates were lower than among those < 5 years and differences in rates between viruses were less pronounced. These findings can help identify age groups at highest risk of hospitalization for each virus and can help guide prevention and treatment strategies.

**Disclosures:**

Melissa Sutton, MD, MPH, Centers for Disease Control and Prevention Emerging Infections Program: Grant/Research Support

